# SABRE: a method for assessing the stability of gene modules in complex tissues and subject populations

**DOI:** 10.1186/s12859-016-1319-8

**Published:** 2016-11-14

**Authors:** Casey P. Shannon, Virginia Chen, Mandeep Takhar, Zsuzsanna Hollander, Robert Balshaw, Bruce M. McManus, Scott J. Tebbutt, Don D. Sin, Raymond T. Ng

**Affiliations:** 1 PROOF Centre of Excellence, Vancouver, BC Canada; 2 Department of Computer Science, UBC, Vancouver, BC Canada; 3 BC Centre for Disease Control, Vancouver, BC Canada; 4 Department of Pathology and Laboratory Medicine, UBC, Vancouver, BC Canada; 5 Department of Medicine, Division of Respiratory Medicine, UBC, Vancouver, BC Canada; 6 UBC James Hogg Research Centre and Institute of HEART + LUNG Health, Vancouver, BC Canada

**Keywords:** Systems biology, Gene modules, Reproducibility, WGCNA, Bootstrap

## Abstract

**Background:**

Gene network inference (GNI) algorithms can be used to identify sets of coordinately expressed genes, termed network modules from whole transcriptome gene expression data. The identification of such modules has become a popular approach to systems biology, with important applications in translational research. Although diverse computational and statistical approaches have been devised to identify such modules, their performance behavior is still not fully understood, particularly in complex human tissues. Given human heterogeneity, one important question is how the outputs of these computational methods are sensitive to the input sample set, or stability. A related question is how this sensitivity depends on the size of the sample set. We describe here the SABRE (**S**imilarity **A**cross **B**ootstrap **RE**-sampling) procedure for assessing the stability of gene network modules using a re-sampling strategy, introduce a novel criterion for identifying stable modules, and demonstrate the utility of this approach in a clinically-relevant cohort, using two different gene network module discovery algorithms.

**Results:**

The stability of modules increased as sample size increased and stable modules were more likely to be replicated in larger sets of samples. Random modules derived from permutated gene expression data were consistently unstable, as assessed by SABRE, and provide a useful baseline value for our proposed stability criterion. Gene module sets identified by different algorithms varied with respect to their stability, as assessed by SABRE. Finally, stable modules were more readily annotated in various curated gene set databases.

**Conclusions:**

The SABRE procedure and proposed stability criterion may provide guidance when designing systems biology studies in complex human disease and tissues.

**Electronic supplementary material:**

The online version of this article (doi:10.1186/s12859-016-1319-8) contains supplementary material, which is available to authorized users.

## Background

Gene network inference (GNI) from whole transcriptome expression data is a fundamental and challenging task in computational systems biology, with potentially important applications in translational research. Often, a major aim is to identify sets of coordinately expressed genes, termed network modules, and to study their properties and how they change across conditions [1–6]. These gene network modules may represent novel, context specific, functional biological units, and identifying and studying such modules has become an important tool of systems biology [7–10]. Although diverse computational and statistical approaches have been devised to identify such modules [11–18], their performance behavior is still not fully understood, particularly in complex human tissues. This situation is understandable – objective assessment is challenging in this context. Nevertheless, these tools are being applied to the study of complex diseases [19, 20], and evaluating the performance, as well as understanding the limitations of state-of-the-art methods in this context, is important. This is particularly true for translational research where studies tend to be smaller, and are often performed in complex tissues and/or highly heterogeneous patient populations. While it is challenging to directly assess the accuracy of inferred networks derived by these methods in real-world expression datasets, the resulting gene modules have properties that may be objectively measured in such data.

One such property is module reproducibility. A comprehensive review of various means of assessing module reproducibility was published by Langfelder et al. [21]. In that study, the authors outlined two broad categories of module preservation statistics: cross-tabulation and network topology derived (including density, connectivity, and separability), and chose to focus on the latter as a means of assessing module reproducibility, in part because of the difficulty of assessing cross-tabulation module preservation statistics in the absence of an independent validation dataset. Re-sampling approaches, such as cross-validation or bootstrapping, have previously been used to overcome this, however [21–23]. Cross-tabulation reproducibility measures are of particular interest because they can be readily applied to a broad range of gene module identification strategies, including popular clustering approaches [24–26] that do not yield a nodes-and-edges network structure and, therefore, cannot be assessed by network topological metrics.

Here, we introduce a flexible strategy for assessing the reproducibility of gene modules that does not rely on network topology and leverages bootstrap re-sampling in place of an independent validation dataset. Even though the idea of using bootstrap re-sampling to evaluate module stability is not new (e.g., [21–23]), a systematic approach to summarize the results obtained across many re-samplings into an easy-to-interpret criterion of module stability, has yet to be described. We demonstrate that the proposed procedure can provide a useful measure of module stability – how sensitive a module’s gene membership is to changes in the set of samples that were used to identify it – in a large (*n* > 200), highly relevant clinical dataset: gene expression profiling of peripheral whole blood, a highly heterogeneous tissue, in chronic obstructive pulmonary disease (COPD), a complex human disease. COPD is a progressive disease, characterized by non-reversible loss of lung function and sporadic worsening of symptoms (shortness of breath, cough, etc.) termed acute exacerbations of COPD (AECOPD). These exacerbations lead to substantial morbidity and mortality [27]. Additionally, some patients experience exacerbation episodes more frequently, and there is interest in understanding the molecular basis, if any, of this phenotype. Since most exacerbations are associated with bacterial or viral respiratory tract infections [28], immune system monitoring using whole blood, transcriptome-wide, gene expression profiling could provide useful insights [25]. We explored this notion by identifying gene network modules from this gene expression data using three popular strategies: weighted gene co-expression analysis (WGCNA) [11], a method based on the partial least squares regression (PLS) technique described by Pihur, Datta and Datta [18], and a clustering-based approach described by Chaussabel et al. [24].

The proposed procedure is more formally defined below (cf. Methods section). Briefly, the ability to recover similar modules across many re-sampled datasets reflects module stability. We propose to estimate gene module stability by looking for concordance between a reference module set derived from the entire data and a large number of comparator module sets derived from re-sampled datasets. Concordance can be determined using some similarity measure; we propose a variation on the Jaccard similarity coefficient, a statistic commonly used to assess the similarity of sets. The stability of a particular module can be estimated by inspecting the distribution of similarity scores across a large number of repeated re-samplings. A schematic representation of the procedure, termed SABRE (**S**imilarity **A**cross **B**ootstrap **RE**-samplings) is shown in Additional file 1: Figure S1.

To facilitate the prioritizing of modules, we introduce a criterion that summarizes the distribution of similarity measures obtained for a particular reference module across bootstrap re-samplings, and explore some useful properties of this criterion. We hypothesized that modules identified by various algorithms, such as WGCNA and the approaches described by Pihur, Datta and Datta, or Chaussabel and others, vary with respect to their stability and that this quality may provide a useful means of prioritizing specific modules for further study. Moreover, changes in module stability across conditions may suggest loss of regulation, which could be of biological interest.

## Methods

### Dataset

We obtained PAXgene blood samples from 238 patients with chronic obstructive pulmonary disease (COPD), who were enrolled in the Evaluation of COPD Longitudinally to Identify Predictive Surrogate Endpoints (ECLIPSE) study [29]. These patients were clinically stable at the time of blood collection and their demographics are summarized in Additional file 2: Table S1.

### RNA extraction and microarray processing

Blood samples for all subjects and timepoints were collected in PAXgene tubes and stored at −80 °C until analysis. Total RNA was extracted using PAXgene Blood RNA Kits (QIAGEN Inc., Germantown, MD, USA), and integrity and concentration determined using an Agilent 2100 BioAnalyzer (Agilent Technologies Inc., Santa Clara, CA, USA). Affymetrix Human Gene 1.1 ST (Affymetrix, Inc., Santa Clara, CA, USA) microarrays were processed at the Scripps Research Institute Microarray Core Facility (San Diego, CA, USA) in order to assess whole transcriptome expression. The microarrays were checked for quality using the RMAExpress software [30] (v1.1.0). All microarrays that passed quality control were background corrected and normalized using quantile normalization (as in RMA) [30] and summarized using a factor analysis model (factor analysis for robust microarray summarization [FARMS]) [31], via the ‘farms’ R package. FARMS includes an objective feature filtering technique that uses the multiple probes measuring the same target transcript as repeated measures to quantify the signal-to-noise ratio of that specific probe set. Informative probe sets, as identified by FARMS (2512), were used for all downstream analyses. Limiting the feature space in this manner had the additional benefit of speeding up identification of gene network modules in the next step, an important consideration given the proposed bootstrapping procedure.

### Identification of gene modules

We identified network modules using three different approaches: weighted gene co-expression network analysis (WGCNA) [11], via the ‘WGCNA’ R package [32], a method based on the partial least squares regression (PLS) technique described by Pihur, Datta and Datta [18], and a k-means clustering-based approach to identifying sets of coordinately expressed transcripts described by Chaussabel and others [24]. In WGCNA, the unsigned gene-gene correlation matrix was weighted to produce a network with approximately scale-free topology, characteristic of biological systems [32]. Average linkage hierarchical clustering is then applied to this weighted gene co-expression network, and the resulting dendrogram cut at a height of 0.15 to produce modules with minimum size of 50. The total number of modules identified in this manner was not fixed. We adopted a similar strategy to identify modules using the approach described by Pihur and others, this time applying average linkage clustering to the gene-gene interaction matrix output from the PLS procedure and cutting the resulting dendrogram at a heaight of 0.15, as before. The Chaussabel approach employs the k-means clustering algorithm to identify co-clustering genes. This algorithm uses a top down strategy in which genes are randomly divided into a predetermined number of clusters. Genes are then iteratively re-assigned to their nearest cluster by some distance function (Euclidean distance, in this case), and cluster centers re-computed under the new configuration. This process is repeated until the algorithm converges. The number of clusters, or gene modules, was necessarily fixed in this case. We used the elbow criterion to determine the point at which the inclusion of additional clusters no longer provides a large increase in proportion of variance explained. Because the initial centers are arbitrarily chosen, the solution is unlikely to be the minimal sum of squares of all possible partitions. Rather a local minimum is returned where any further reassignment of a gene from one cluster to another will not reduce the within cluster sum of squares. To address this we used 10 random initial configurations and retained the solution with minimal sum of squares. Although this provides a minimum over several partitions, it does not guarantee a global minimum solution.

### Assessing the stability of gene modules

All three approaches were applied as described to all 238 peripheral whole blood expression profiles to identify modules of coordinately expressed transcripts. These are termed the reference module sets for each approach. To assess stability of all modules within the reference module sets, bootstrap re-sampling was used to generate 1000 random sets of 238 subjects. All three network module identification approaches were applied to all re-samplings to produce 1000 bootstrapped sets of modules, in each case.

In order to describe how we propose to assess the stability of the reference modules, we must first introduce a few related concepts. We start with the concept of module *accuracy*, defined as follows: for each reference module *q*, a set of genes of size |*q*|, and a test module *q*′, where |*q* ∩ *q*′| is the number of genes in common between the two. Accuracy is then defined as:

1 $$ Accurac{y}_q=\frac{\left|q{\displaystyle \cap }{q}^{\prime}\right|}{\left|q\right|}\in \left[0,1\right] $$

We instead propose to use the closely related Jaccard similarity coefficient, a statistic commonly used to assess the similarity of sets:

2 $$ Jaccar{d}_{q{q}^{\prime }}=\frac{\left|q{\displaystyle \cap }{q}^{\prime}\right|}{\left|q{\displaystyle \cup }{q}^{\prime}\right|}\in \left[0,1\right] $$

Comparison of a reference module to a test module that is an exact subset should be considered a perfect match from a stability standpoint (the similarity measure used should return 1 in this case). In order for the similarity measure used to reflect this, we modify (2) as follows:

3 $$ Similarit{y}_{q{q}^{\prime }} = \frac{\left|q{\displaystyle \cap }{q}^{\prime}\right|}{min\left\{\left|q\right|,\ \left|{q}^{\prime}\right|\right\}}\ \in\ \left[0,\ 1\right] $$

This is analogous to the Simpson index that can be computed for bipartite networks [33], but note that similarity is defined only in terms of the degree of overlap between modules members or nodes. The modules are treated as simple sets in (3).

Finally, gene module stability can be very naturally estimated via a random re-sampling with replacement procedure, termed bootstrapping [34], by looking for concordance between a reference module set derived from the entire data and one derived from a re-sampled dataset. Concordance can be determined using the similarity measure defined above (3). When comparing a module *q* to a module set *Q* = {*q*_1_, *q*_2_, *q*_3_,…,*q*_*n*_}, the best match for *q* is defined to be *q*′, such that (3) between *q* and *q*′ is the highest among all possible comparisons between *q* and members of *Q*. The best match similarity coefficient is then (3) between *q* and *q*′, and the stability of a particular module *q* can be estimated by looking at the distribution of best match similarity scores across a large number of repeated re-samplings, {*R*_*j*_; *j* = 1,2,…,*n*}. In order to rank modules by their stability, we summarize these distributions for each module to a single number, the Hirsch index (H-index), as follows:

4 $$ H- index(q) = { \max}_h\left\{\left[\frac{1}{1000}{\displaystyle {\sum}_{j=1}^{1000}}\left({R}_j\ge h\right)\right]\ge h\right\}\in \kern0.75em \left[0,\ 1\right] $$

For a reference module with H-index = 0.8, 0.8 similarity or greater was observed in 80 % of bootstrap runs. A more qualitative interpretation would be that we expect this reference module, derived from all available samples, to have 80 % similarity to a hypothetical module derived from a whole population dataset.

### Construction of random gene modules

To get a sense of the stability that could be expected of a module containing genes with minimal relation to each other, we carried out a simulation study. Modules of size 50–400 (by increments of 50) were created by sampling from the all 2512 gene symbols in the FARMS filtered dataset. This was done 100 times for each size of module. Then, for each of these random modules, the best match Jaccard similarity coefficient was recorded across all 1000 module sets generated during the previously described bootstrap procedure. The resulting distribution was summarized using the H-index.

### Stability of network modules, sample size, and module size

In order to study the relationship between network module stability, sample size, and module size, a slight variation of the bootstrap re-sampling strategy was used. We randomly sampled, without replacement 10, 20, 40, 80, 120, or 160 expression profiles from the 238 peripheral whole blood expression profiles described above. In each case, a reference module set was produced, 100 bootstrap re-samplings of the selected expression profiles generated, and the stability of the reference module set across bootstrap re-samplings determined as before. This was repeated 10 times to capture the effect the original selection had on generation of the reference module set.

### Stability of network modules and network topology

The relationship between module stability and various network topology measures was also of interest. We constructed an undirected network using the ‘igraph’ R package [35]. We defined a gene-gene edge as that where the absolute correlation for that gene pair was at least two standard deviations away from the mean correlation observed across all possible gene pairs. Various topology measures were then calculated for each of the reference modules: average number of neighbors per gene (divided by module size), number of instances in which a gene appears in a shortest path between two other genes, and number of triads (divided by number of possible triads in the module), and compared to their stability.

### Stability of network modules and functional annotation

Finally, we explored the relationship between stability and our ability to functionally annotate gene modules. We hypothesized that stable or reproducible gene modules should correspond well to known biological programs more often than unstable ones. To test this, we compared the gene membership of our reference modules to the MSigDB collections (v5.0) [36]. We also included the recently described Blood Transcriptome Modules (BTMs), a collection of blood-specific transcriptomic modules derived from an analysis of over 30,000 human blood transcriptome profiles from more than 500 studies whose data are publicly available [26]. Annotation was done by testing for over-representation of genes from the MSigDB gene sets using a hypergeometric test, a simple statistical method commonly used to quantitatively measure enrichment [37]. To quantify how well a particular module was annotated in the gene set collection, we computed the sum of the –log_10_ of the *p*-values for the hypergeometric test across all gene sets in the collection and compared it to its stability, separately in each of the collections (MSigDB Hallmark, C1-C7, and BTMs).

## Results

We first identified sets of gene modules by using all available samples and by employing three different approaches: weighted gene co-expression network analysis (WGCNA) [11], a method based on the partial least squares regression (PLS) technique described by Pihur, Datta and Datta [18], and a clustering-based approach to identifying sets of coordinately expressed transcripts described by Chaussabel and others [24]. These are termed reference module sets.

When applied to all 238 samples, WGCNA identified 19 network modules, ranging in size from 69 to 850 probe-sets (36 to 427 genes; Additional file 3: Table S2), with a mean module size of 240 probe-sets (median = 157). The method from Pihur, Datta and Datta, identified 24 network modules, ranging in size from 69 to 554 probe-sets (32 to 210 genes; Additional file 4: Table S3), with a mean module size of 207 probe-sets (median = 157). The Chaussabel approach identified 20 network modules ranging in size from 44 to 302 probe-sets (38 to 167 genes; Additional file 5: Table S4), with mean module size of 131 probe-sets (median = 120). Modules identified by WGCNA and the Pihur approach were highly concordant (mean Jaccard similarity = 0.78; not shown), while those identified by the Chaussabel approach were largely distinct (Jaccard similarity < 0.2 for the majority of module pair-wise comparisons; Additional file 6: Figure S2) with the exception of the WGCNA-derived turquoise and blue modules, which were similar to the Chaussabel-derived M17 and M19 modules, respectively (similarity coefficient > 0.5). Annotation of the turquoise/M17, and blue/M19 modules, was consistent (Additional file 7: Table S5).

### Assessing gene module stability using SABRE and the H-INDEX

**Fig. 1:**
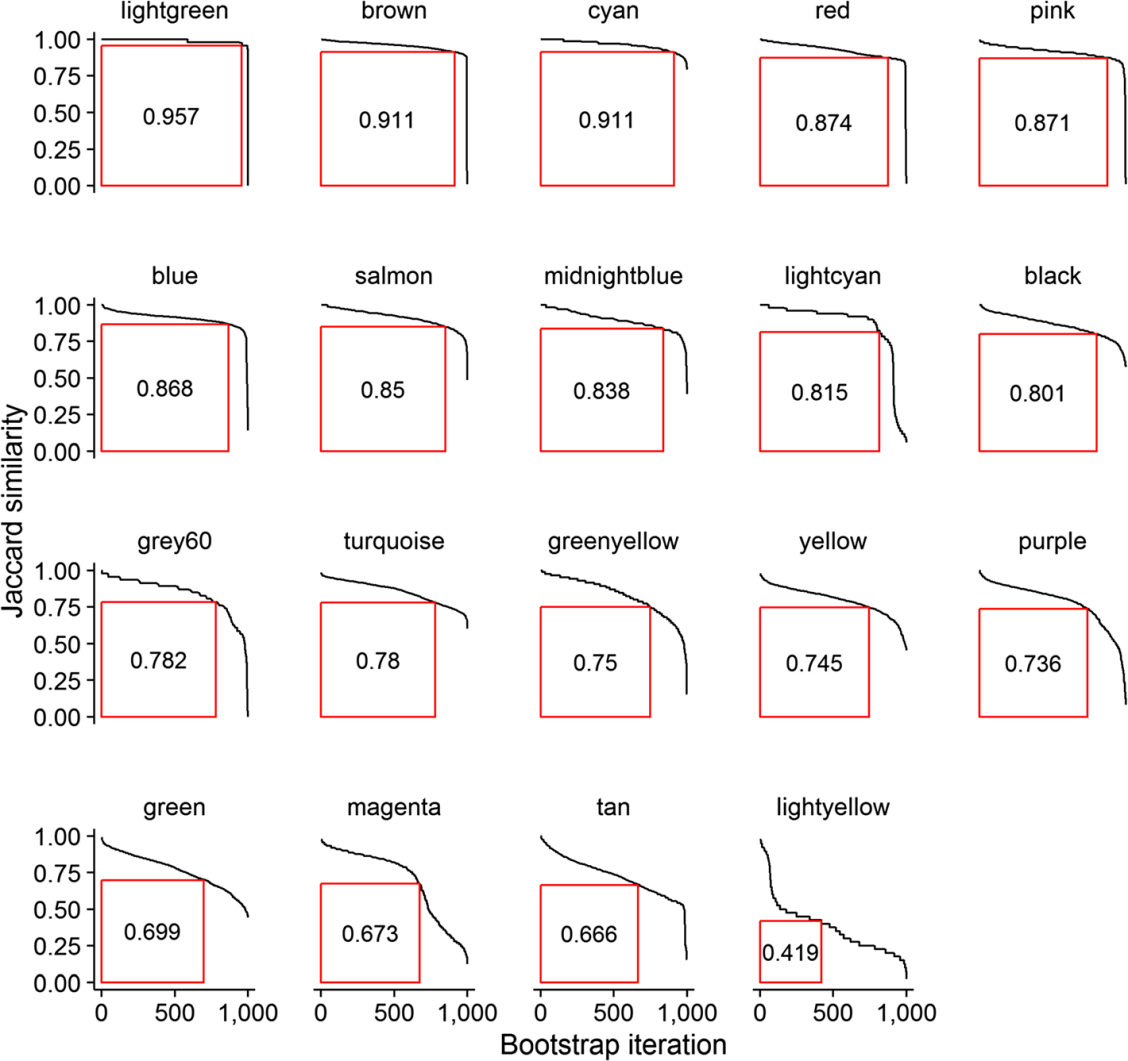
Visual derivation of the H-index. A visual depiction of the derivation of the H-index is shown for modules identified by the WGCNA algorithm. A set of reference modules derived from all available samples are compared to a series of comparator module sets derived from bootstrapped re-sampled data. For each re-sampled dataset, all reference modules are compared to all newly identified modules using the Jaccard similarity coefficient. For each reference module, the best match Jaccard similarity coefficient value is recorded. Finally, these best match similarity coefficients are sorted and a measure of the area under the resulting curve (Hirsch-index) used to estimate the reference module’s stability

Next, we applied SABRE to assess the stability of these reference modules. Refer to Methods for detail. Briefly, each algorithm was allowed to identify a set of network modules from each re-sampling and these re-sampled module sets were compared to the reference module set. For each reference module, only the highest observed similarity coefficient within each re-sampling (that for the best matching re-sampled module) was recorded and the distribution of these similarity coefficients across all bootstrap re-samplings was used to assess stability. Modules with distribution of similarity coefficients across all re-samplings that skewed towards one were consistently matched to highly concordant re-sampled modules across many random sample configurations and are thus deemed to be relatively insensitive to sample outliers. To simplify interpretation, we summarized the distribution of similarity coefficients for each module to its Hirsch-index [38] (H-index), as defined in (4). A visual derivation of the H-index is shown in Fig. 1 for the modules identified by WGCNA.

### Gene module stability increases with sample size and module size

**Fig. 2:**
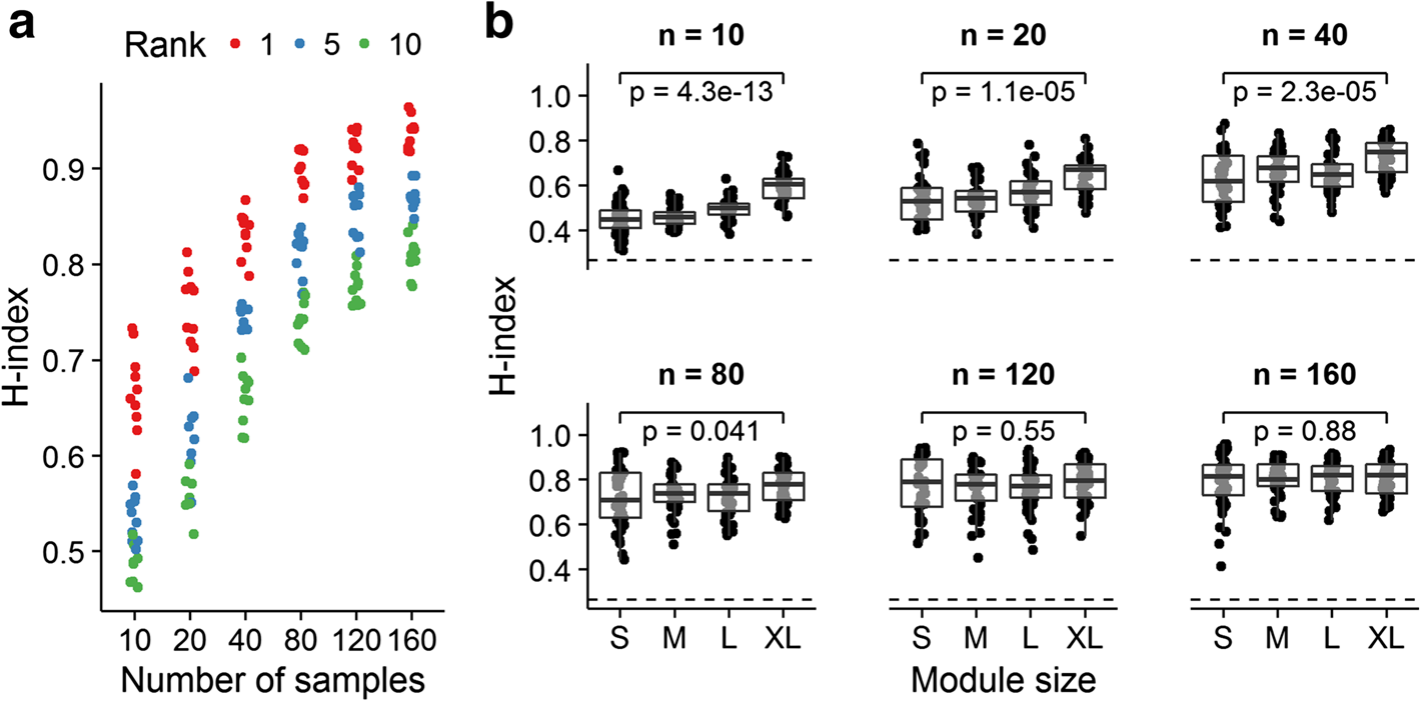
Gene module stability increases with sample size and module size. For each n, we sampled without replacement from all available gene expression profiles 10 times. In each case, a reference module set was produced (by WCGNA), 100 bootstrap re-samplings of the selected expression profiles generated, and the stability of the reference module set across bootstrap re-samplings determined as described in the Methods section. **a** Stability of the modules is visualized at *n* = 10, 20, 40, 80, 120 and 160 for the 1st, 5th and 10th rank modules. **b** Stability is plotted against module size at *n* = 10, 20, 40, 80, 120 and 160. The dotted line depicts the best-case stability of random modules in simulation. We compare the stability of S (1^st^ quartile) and XL (4^th^ quartile) modules using Wilcoxon’s rank-sum test

Gene modules are often identified in relatively small study populations. We used a variation of the SABRE strategy to study the effect of sample size on gene module stability. As expected, module stability, as assessed using the current framework, increased as the sample size was increased (Fig. 2a). This relationship held for both very stable (1^st^ rank) and less stable (10^th^ rank) modules. We also observed a relationship between module stability and module size in smaller studies (Fig. 2b; *n* = 10, *p* = 4.3 × 10^−13^; *n* = 80, *p* = 0.041; Wilcoxon’s rank-sum test), but there was no such relationship at larger sample sizes (*n* = 120, *p* = 0.55; *n* = 160, *p* = 0.88). In all cases, modules identified (by WGCNA in this case) were more stable than modules assembled by randomly sampling from all gene symbols in the filtered dataset, even when sample size was small (Additional file 8: Figure S3).

### Stability profiles differ between algorithms

**Fig. 3:**
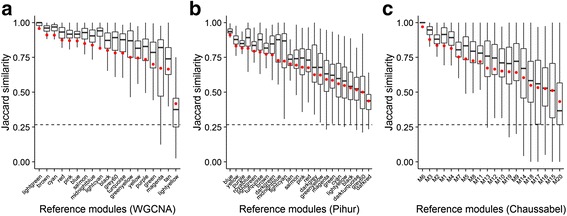
Stability profiles differ between algorithms. Gene module similarity across bootstrap re-samplings, for all reference network modules identified by three gene module discovery algorithms, is visualized using box plots (**a**: WGCNA; **b**: Pihur, **c**: Chaussabel). The stability of the network modules is summarized using the H-index (red). The dotted line depicts the best-case stability of random modules in simulation

The H-index was next used to rank reference modules (Fig. 3). Modules varied with respect to their stability as assessed by the SABRE procedure, both within sets of modules identified by particular algorithms, as well as across strategies. Modules identified by all three strategies were significantly more stable than modules assembled by randomly sampling from all gene symbols in the filtered dataset (Additional file 8: Figure S3). The set of network modules identified by WGCNA was more stable (mean H-index = 0.79, standard deviation = 0.12, range = 0.42–0.96) than that identified by either the Pihur (mean H-index = 0.68, standard deviation = 0.12, range = 0.44–0.91), or Chaussabel approaches (mean H-index = 0.69, standard deviation = 0.14, range = 0.43–0.97), though the top ranking module identified by all three approaches had comparable stability (WGCNA: lightgreen module, H-index = 0.96; Pihur: blue module, H-index = 0.91; Chaussabel: M6 module, H-index = 0.97).

### Stable modules are more interconnected

**Fig. 4:**
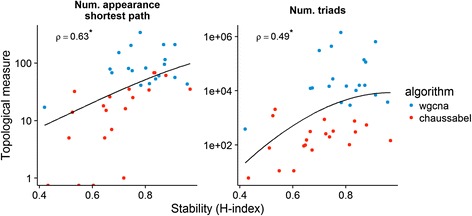
Stable modules are more interconnected. The relationship between module stability and a number of topological measures of network connectivity is visualized for modules identified by WGCNA (blue) or the Chaussabel approach (red). Stability is positively associated (Spearman’s ρ) with both number of appearance in the shortest path and number of triads in the network (* *p* ≤ 0.05)

We might expect more connected gene network modules to be more stable. There are many proposed metrics for measuring network connectivity. The simplest such metric is the average number of neighbors. For our dataset, there is no observed trend between the H-index and the average number of neighbors within a module. A stronger notion of connectivity is to measure the number of nodes that are in the shortest path between some pair of nodes in the network. The higher this number, the more tightly connected the network. Figure 4 suggests that there is a relationship between this notion of connectivity and the H-index. An even stronger notion of connectivity described in the literature is the number of triads, which are triplets of nodes that are directly connected pairwise. Figure 4 indicates that there is a weak relationship between this notion of connectivity and the H-index, particularly for lower values of the H-index. In sum, it appears that the notion of stability indicated by the H-index is related, but not identical, to 2 well-known notions of connectivity in network science.

### Stable modules are more readily annotated

**Fig. 5:**
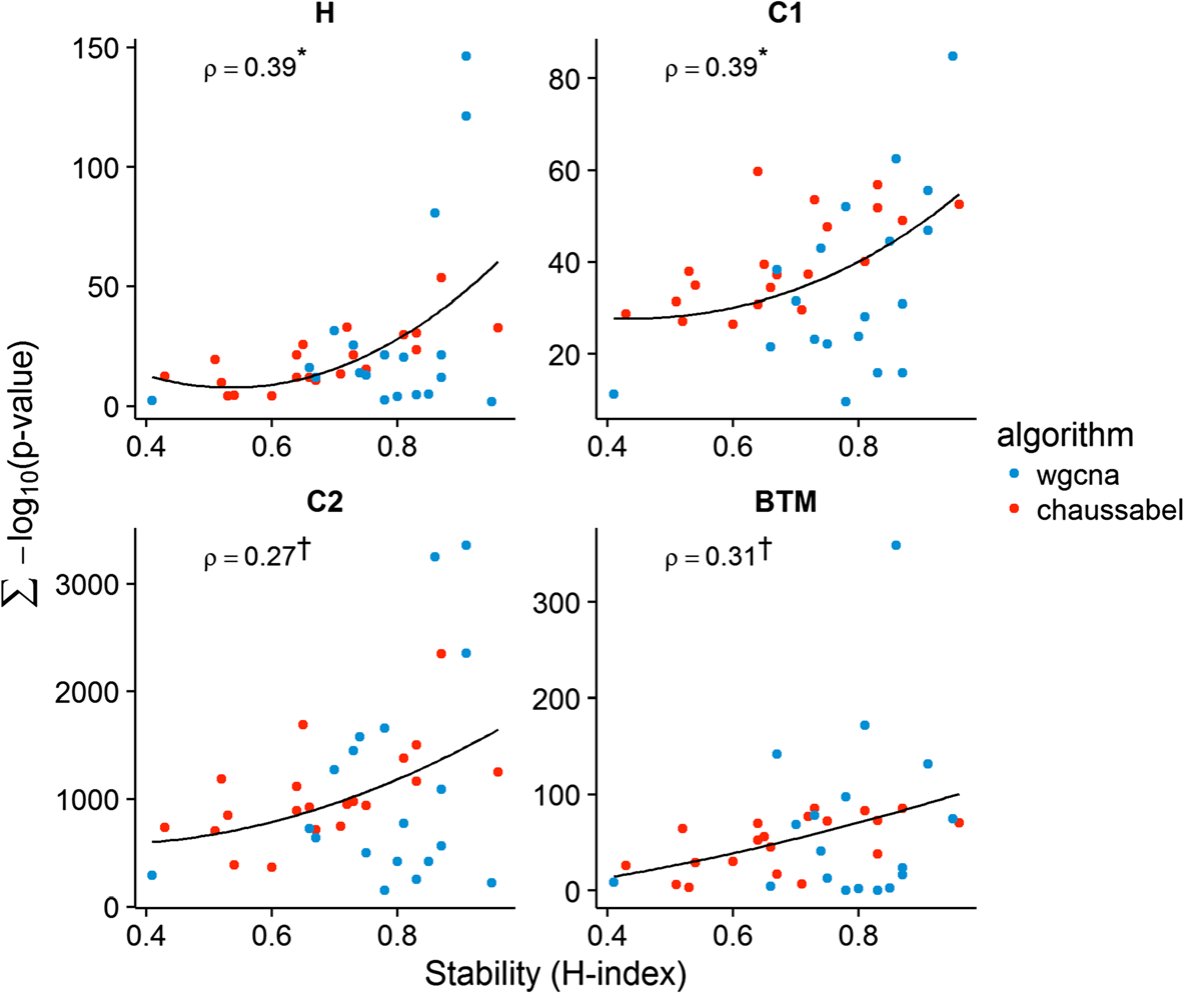
Stable modules are more readily annotated. Module gene over-representation in annotated gene sets (sum of –log_10_ *p*-value for the hypergeometric test) is visualized, for modules with varying stability, in the MSigDB and BTM collections. Stability is positively associated (Spearman’s ρ) with our ability to assign module to known biology (* *p* ≤ 0.05; † *p* ≤ 0.10) in many of these collections

We hypothesized above that stable modules should correspond to well-characterized biological functions. If this is true, we would expect stable modules to be more readily annotated than less stable ones. We explored this notion in the BTM and MSigDB collections of annotated gene sets and found that stable modules were indeed more readily annotated across many of the included collections (Fig. 5). Module stability was significantly associated with annotatability (sum –log_10_ *p*-value of the hypergeometric test of the overlap between annotation and module genes) in the hallmark (H, Spearman’s *ρ* = 0.39; *p* = 0.01), positional (C1, Spearman’s *ρ* = 0.39; *p* = 0.01), immunologic signatures (C7, Spearman’s *ρ* = 0.31; *p* = 0.05), and blood transcriptomic modules (BTM, Spearman’s *ρ* = 0.31; *p* = 0.05) collections, marginally associated in the canonical (C2, Spearman’s *ρ* = 0.27; *p* = 0.09), and oncogenic signatures (C6, Spearman’s *ρ* = 0.27; *p* = 0.10) collections, and showed no significant association in the gene ontology (GO, C5), motif (C3), or computational (C4) gene set collections. Complete gene set over-representation results are tabulated in Additional file 5: Table S4.

Many gene modules identified by WGCNA had very high concordance with gene sets corresponding to distinct biological functions (*e.g*. lightgreen: B cell activity, antibody production; cyan: interferon signaling; brown: heme metabolism; blue: recruitment of neutrophils and TLR mediated inflammatory signaling). In fact, even relatively less stable modules identified by WGCNA appeared to correspond to known biological pathways (*e.g*. purple module [H-index = 0.73]: MHC class II antigen presentation). Modules identified by the Chaussabel approach had generally lower concordance to annotated gene sets, with modules often having seemingly overlapping biological function (*e.g*. interferon signaling pathway activity was assigned to both modules M04 and M06). In fact, nearly half of gene modules identified by the Chaussabel approach were enriched in genes associated with translation (7/20 modules had significant enrichment with KEGG ribosome, KEGG translation) and mRNA transcription (4/20 modules had significant enrichment with KEGG spliceosome, REACTOME metabolism of mRNA, REACTOME Translation, REACTOME mRNA splicing).

## Discussion

The main objective of this work was to devise a strategy for evaluating the stability of gene modules in a manner applicable to a broad range of gene module identification strategies, and not reliant on the availability of additional datasets for validation. Quantifying network module stability would allow for prioritization of modules for further experimental interrogation. While the use of re-sampling strategies, such as cross-validation or the bootstrap, in this context has been previously proposed [21–23], a systematic approach to summarizing the results obtained across many re-samplings into an easy-to-interpret criterion of module stability, has yet to be described. As shown in Fig. 1, for a particular module, all the re-sampling results can be captured by a curve. The question we ask is how to summarize the curve with a single value that is informative. Standard ways, such as using the mean value, or even using the area-under-the-curve value, do not provide a “minimum quality” guarantee. In contrast, the proposed bootstrap re-sampling and summarization scheme, SABRE, does provide such a lower bound guarantee, at least across all generated bootstrap runs. The proposed stability criterion, the H-index of the curve, corresponding to the largest square under the curve, is readily interpreted: for a module with H-index = 0.8, one can say that similarity of 0.8 or greater was observed across at least 80 % of the bootstrap runs.

We go on to explore a number of useful characteristics of the H-index. We show that the H-index of a module generally increases as sample size increases and observe that, for any given module, a stability maximum appears to be reached between *n* = 80–120, at least in this tissue and patient population. We also note that, while smaller modules are generally less stable, this is not the case with larger sample sizes (*n* > 80–120). As expected, we find randomly assembled modules to have very low stability (H-index = 0.25), and it is comforting to note that, even for very small studies (*n* = 10), modules identified by WGCNA had worst case stability that exceeded this value, suggesting that, for some gene modules at least, core members may be identifiable even in very small studies. Taken together, these observations suggest that the identification of robust gene expression modules in complex tissues and diseases requires large study populations (*n* > 100).

Next, we compare gene network modules identified by three popular gene network module identification strategies. WGCNA and the PLS-based approach described by Pihur, Datta and Datta identified largely concordant sets of gene network modules, while the modules identified by the Chaussabel approach were largely distinct. Given that module identification for both WGCNA and the Pihur approach utilized average linkage hierarchical clustering on an adjacency matrix, this is perhaps not surprising. Applying SABRE to these modules sets allowed us to readily rank identified gene modules from most to least stable. Modules identified by WGCNA were generally more stable than those identified by the other two approaches. The ranking of modules by their stability, in combinations with other metrics, such as biological annotation, may inform prioritization of certain modules for further study.

We found that the H-index was positively associated with topological notions of network connectivity, as well as our ability to assign biological function to gene modules. SABRE uncovered important qualitative differences between module sets in this respect, however. First, while stability was positively correlated with connectivity across all modules, this relationship was strongest in modules identified by the Chaussabel approach. These modules also exhibited lower network connectivity compared to those found by WGCNA. This is not surprising since the Chaussabel approach pays no attention to the topology of the constructed modules. A similar pattern emerges when comparing the relationship between module stability and annotatability in the MSigDB and BTM gene set collections. Here again, the relationship between stability and annotatability was strongest in modules identified by the Chaussabel approach. Given the distribution of the stability criterion, and the connectivity or annotatability measures considered, it is difficult to determine whether the observed relationships between stability and connectivity/annotatability are true or primarily driven by broad differences in stability between module sets identified by different strategies.

Stable gene modules that do not readily correspond to annotated gene sets may be very interesting, of course, as they may represent novel, disease- or tissue-specific biological processes. Two such gene modules were identified in the current study: the lightgreen (h = 0.96) and blue (h = 0.86) modules. In an effort to assign biological function to these modules, we compared them to the recently published Blood Transcriptome Modules (BTM). These empirically derived sets of co-regulated genes have very low overlap with presently available pathways and were identified in peripheral whole blood gene expression data. We reasoned that the highly stable, un-annotated modules we independently identified in this study may in fact correspond to some of these highly stable blood modules. In fact, this was the case: the lightgreen module was enriched for a number of BTMs related to B cell activity, while the blue module was matched to various innate immunity BTMs (recruitment of neutrophils and TLR mediated inflammatory signaling). Both B cells and neutrophils are known to be implicated in COPD. [39, 40] This provided validation, both of the modules themselves, and of the stability ranking produced by the SABRE procedure, in that two highly stable modules that did not correspond to any available pathway annotations, were consistent with independently derived functional modules specific to blood leukocyte sub-populations. These modules may represent important and novel biological function in the peripheral whole blood compartment of COPD patients.

## Conclusions

In conclusion, we demonstrate that bootstrap re-sampling, and the SABRE procedure described herein, can assess the stability of gene modules identified by three different algorithms and suggest that this could be a useful criterion when selecting modules for further investigation. We also show that when modules are identified in smaller studies, more stable ones are more likely to replicate in larger experiments compared to less stable ones. We show a relationship between this notion of stability, topological connectivity, and our ability to assign biological function to gene modules. Our approach highlights the relative robustness of the WGCNA algorithm to sample outliers and, more generally, suggests that many gene module strategies should probably be applied jointly to any given dataset. Finally, we identify and validate two highly stable modules that may represent novel, tissue-specific biological function in the context of the peripheral whole blood of clinically stable COPD patients.

## Additional files

Additional file 1: Figure S1. Schematic of bootstrap re-sampling procedure. (PNG 146 kb)
Additional file 2: Table S1. Patient demographics. Demographic characteristics of the 238 COPD patients. (CSV 495 bytes)
Additional file 3: Table S2. WGCNA reference module sets. (CSV 550 bytes)
Additional file 4: Table S3. Pihur reference module sets. (CSV 701 bytes)
Additional file 5: Table S4. Chaussabel reference module sets. (CSV 497 bytes)
Additional file 6: Figure S2. WGCNA and Chaussabel module concordance. Concordance between network modules identified by two gene network module discovery. Reference module sets were identified using all available gene expression profiles. The similarity coefficient for each pair-wise comparison is visualized as a clustered heatmap, with red indicating high similarity and blue indicating low similarity. (PNG 77 kb)
Additional file 7: Table S5. Pathway over-representation analysis results for the WGCNA and Chaussabel modules sets. For each module identification strategy and module in turn, we carried out pathway over-representation analysis against the Broad Institute’s MSigDB collections by comparing the gene set and module membership using a simple hypergeometric test. We report both *p*-value and false discovery rate adjusting for multiple comparisons using the Benjamini-Hochberg procedure. (CSV 196 kb)
Additional file 8: Figure S3. Random module stability. To get a sense of the stability that could be expected of a module containing genes with minimal relation to each other, a simulation study was carried out. Modules of size 50, 100, 150, 200, 250, 300, 350, and 400 were randomly assembled by sampling from the all 2512 gene symbols in the filtered dataset. This was done 100 times for each size of module. For each random module, their best match Jaccard similarity ceofficients were computed for each of the 1000 bootstrap results previously generated, and the resulting distribution was summarized using the h-index. (PNG 51 kb)

## Abbreviations

AECOPD: Acute exacerbation of chronic obstructive pulmonary disease
COPD: Chronic obstructive pulmonary disease
GNI: Gene network inference
SABRE: Stability across bootstrap re-samplings
WGCNA: Weighted gene co-expression analysis
