# Supplementary figures and images for: SABRE: a method for assessing the stability of gene modules in complex tissues and subject populations
Source: BMC Bioinformatics. 2016 Nov 14;17:460. doi: 10.1186/s12859-016-1319-8 (PMC5109843; doi:10.1186/s12859-016-1319-8)

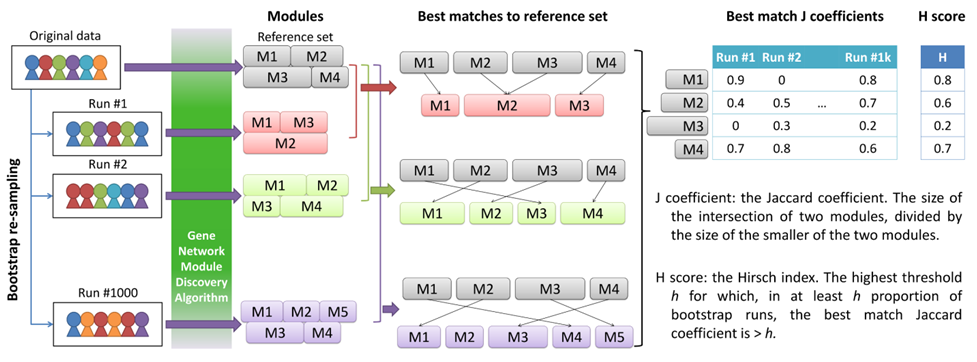

Supplement: Additional file 1: Figure S1. — Schematic of bootstrap re-sampling procedure. (PNG 146 kb) [file 12859_2016_1319_MOESM1_ESM.png]

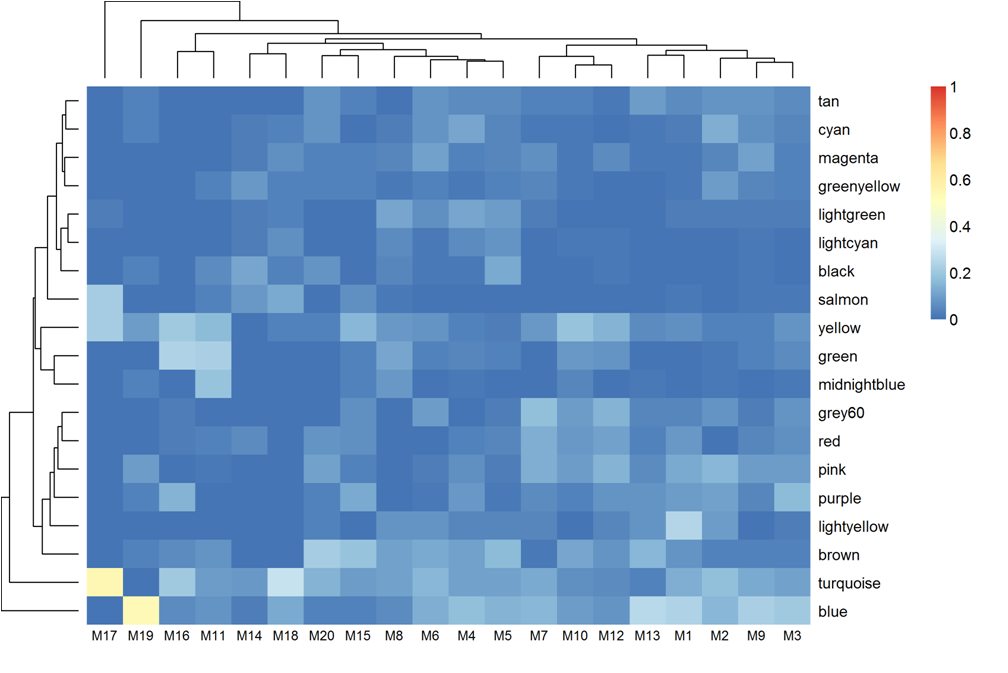

Supplement: Additional file 6: Figure S2. — WGCNA and Chaussabel module concordance. Concordance between network modules identified by two gene network module discovery. Reference module sets were identified using all available gene expression profiles. The similarity coefficient for each pair-wise comparison is visualized as a clustered heatmap, with red indicating high similarity and blue indicating low similarity. (PNG 77 kb) [file 12859_2016_1319_MOESM6_ESM.png]

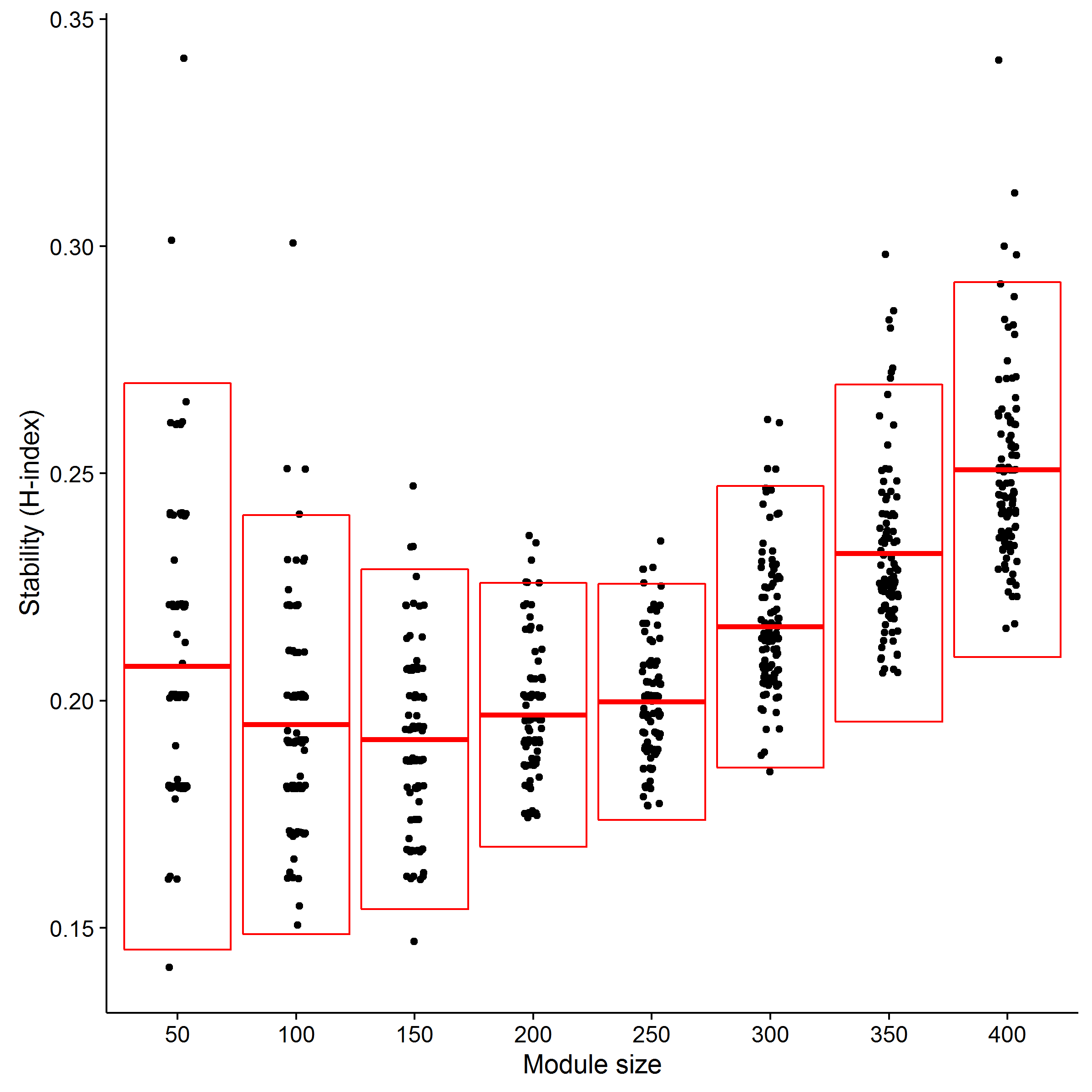

Supplement: Additional file 8: Figure S3. — Random module stability. To get a sense of the stability that could be expected of a module containing genes with minimal relation to each other, a simulation study was carried out. Modules of size 50, 100, 150, 200, 250, 300, 350, and 400 were randomly assembled by sampling from the all 2512 gene symbols in the filtered dataset. This was done 100 times for each size of module. For each random module, their best match Jaccard similarity ceofficients were computed for each of the 1000 bootstrap results previously generated, and the resulting distribution was summarized using the h-index. (PNG 51 kb) [file 12859_2016_1319_MOESM8_ESM.png]
